# Optimized Replacement T4 and T4+T3 Dosing in Male and Female Hypothyroid Patients With Different BMIs Using a Personalized Mechanistic Model of Thyroid Hormone Regulation Dynamics

**DOI:** 10.3389/fendo.2022.888429

**Published:** 2022-07-14

**Authors:** Mauricio Cruz-Loya, Benjamin B. Chu, Jacqueline Jonklaas, David F. Schneider, Joseph DiStefano

**Affiliations:** ^1^ Department of Computational Medicine, David Geffen School of Medicine, University of California, Los Angeles, Los Angeles, CA, United States; ^2^ Division of Endocrinology, Georgetown University, Washington, DC, United States; ^3^ Department of Surgery, Division of Endocrine Surgery, University of Wisconsin, Madison, WI, United States; ^4^ Department of Computer Science, University of California, Los Angeles, Los Angeles, CA, United States; ^5^ Department of Medicine, University of California, Los Angeles, Los Angeles, CA, United States

**Keywords:** simulation, math model, monotherapy, combination hormone therapy, residual thyroid function, p-THYROSIM, anthropometric parameters, personalized therapy

## Abstract

**Objective:**

A personalized simulation tool, p-THYROSIM, was developed (1) to better optimize replacement LT4 and LT4+LT3 dosing for hypothyroid patients, based on individual hormone levels, BMIs, and gender; and (2) to better understand how gender and BMI impact thyroid dynamical regulation over time in these patients.

**Methods:**

p-THYROSIM was developed by (1) modifying and refining THYROSIM, an established physiologically based mechanistic model of the system regulating serum T3, T4, and TSH level dynamics; (2) incorporating sex and BMI of individual patients into the model; and (3) quantifying it with 3 experimental datasets and validating it with a fourth containing data from distinct male and female patients across a wide range of BMIs. For validation, we compared our optimized predictions with previously published results on optimized LT4 monotherapies. We also optimized combination T3+T4 dosing and computed unmeasured residual thyroid function (RTF) across a wide range of BMIs from male and female patient data.

**Results:**

Compared with 3 other dosing methods, the accuracy of p-THYROSIM optimized dosages for LT4 monotherapy was better overall (53% vs. 44%, 43%, and 38%) and for extreme BMI patients (63% vs. ~51% low BMI, 48% vs. ~36% and 22% for high BMI). Optimal dosing for combination LT4+LT3 therapy and unmeasured RTFs was predictively computed with p-THYROSIM for male and female patients in low, normal, and high BMI ranges, yielding daily T3 doses of 5 to 7.5 μg of LT3 combined with 62.5–100 μg of LT4 for women or 75–125 μg of LT4 for men. Also, graphs of steady-state serum T3, T4, and TSH concentrations vs. RTF (range 0%–50%) for untreated patients showed that neither BMI nor gender had any effect on RTF predictions for our patient cohort data. Notably, the graphs provide a means for estimating unmeasurable RTFs for individual patients from their hormone measurements before treatment.

**Conclusions:**

p-THYROSIM can provide accurate monotherapies for male and female hypothyroid patients, personalized with their BMIs. Where combination therapy is warranted, our results predict that not much LT3 is needed in addition to LT4 to restore euthyroid levels, suggesting opportunities for further research exploring combination therapy with lower T3 doses and slow-releasing T3 formulations.

## Introduction

Screening for hypothyroidism in patients is based largely on serum (TSH) concentrations, and patients with TSH levels in the normal range (approximately 0.4 to 4 mU/L) are typically assumed to have normal thyroid function. When TSH levels exceed the upper limit of this range, patients are candidates for further testing for hypothyroidism [for example, by having their serum free T4 (FT4) levels checked]. If diagnosed with hypothyroidism, they are typically prescribed oral levothyroxine (LT4) replacement treatment (1). For patients believed to have minimal endogenous thyroid function, the initial prescribed LT4 dose is often computed using the simple body weight (BW) formula 1.6 μg LT4 per kg body weight (BW) (2), and subsequently adjusted as needed until a TSH plasma concentration in the normal range is achieved—assumed to indicate restoration of euthyroidism.

Precise LT4 dosing is complicated by differences in BW and other measures of body dimensions, as well as confounding physiological responses to exogenous LT4 treatment. In euthyroid individuals, serum TSH increases and serum T4 decreases with increasing body mass index (BMI) (3–5). Some studies have found that serum T3 concentrations remain roughly constant (3), while others have found an increase in T3 and the T3/T4 ratio with increasing BMI (6). Notably, thyroid hormone metabolism and treatment with LT4 also influence body composition; treated hypothyroid patients lose weight due to removal of water accumulated in tissues (myxedema), rather than a decrease in adipose tissue (7,8).

It has become clear that precise dosing in individual patients depends on body composition—characterized by anthropometric parameters such as BMI (2, 9, 10), lean body mass (11, 12), body surface area (13), sex, and age (2,10). A recent review of various dosing algorithms that aim to find an optimal dosing scheme for LT4 replacement therapy reported that accurate dosing indeed depends to some extent on patient BW, height = H (or BMI = H^2^/BW), age, gender, and pre-operative TSH serum concentrations (14). This is an ongoing research area, and a better understanding of thyroid hormone regulation dynamics as a function of these parameters in different patient subgroups can help improve dosing accuracy.

Relying exclusively on TSH values to monitor hypothyroidism and determine monotherapy LT4 replacement dosages has been recently challenged (15, 16). For example, it has been reported that LT4 doses that achieve normal range serum TSH levels often fail to restore serum T4:T3 ratios to the euthyroid normal range (16). For example, a ratio of FT4 x100/T3 is approximately 0.85 in endogenous euthyroidism, but increases to 1.15 with LT4 replacement (17). Also, some patients remain unsatisfied (not restored to their baseline) with LT4 replacement monotherapy. As an alternative to monitoring TSH only, an individualized approach that simultaneously considers FT4, T3, and TSH serum concentrations to optimize dosing has been proposed (15).

In this paper, we approach this hormone replacement problem by incorporating sex, weight, and height differences, as well as hormone levels in individual patients, into a new model of thyroid hormone regulation dynamics. To accomplish this, we modify and expand THYROSIM—our mechanistic physiologically based model of the system regulating serum thyroid hormone and TSH levels, quantified from normal human data for mixed male–female normal weight patients (18–20). We call this new model p-THYROSIM (*personalized* THYROSIM).

Using our model to optimize dosing in this variety of patients, we explored the efficacy of various dosing strategies, first with LT4-only (monotherapy) dosing, using only serum TSH and then both serum TSH and T4 as endpoints. To accomplish this, we quantified our new model with two different experimental datasets (21,22) containing data from distinct male and female patients across a wide range of BWs and heights. These patient populations incorporated 3 different sets of circumstances in the model: healthy volunteers with endogenous thyroid function, hypothyroid patients with some endogenous thyroid function, and thyroidectomized patients. For validation, we compared our predictions for optimized LT4 monotherapies with a third dataset (14), previously published data on optimized LT4 therapies based on various individual patient criteria.

Second, in place of LT4 monotherapy, we explored combination LT4+LT3 replacement therapy, proposed to better emulate normal hormone production and restore the T4:T3 ratios to euthyroid levels (16). Studies evaluating mixed or “combination” therapies have typically obtained mixed or inconclusive results (23–25), likely due in part to lack of consensus on optimal dosing with LT4+LT3, pharmacokinetics of LT3, as well as lack of knowledge or measurement of residual thyroid function (RTF) in hypothyroid patients (26). The original THYROSIM (developed for mixed male–female normal weight patients) was previously used to simulate a wide range of RTF values in typical hypothyroid patients with reduced thyroid hormone secretion rates and their simulated responses to practical combinations of optimized LT3 and LT4 doses (26). We extend those studies here, for both male and female patients separately across a variety of anthropometric parameters.

## Methods and Data

### Patient-Dependent Changes in Plasma Volume, TSH Distribution Volume, and T3 Clearance

To incorporate the effect of an individual’s height, body weight and sex, we mainly model how these features affect plasma volume (*V_P_*), TSH distribution volume (*V_TSH_*), and fractional T3 clearance rate of the patient. Our modifications to plasma volume are derived from an ideal weight formula fitted to a dataset containing 80 men and 80 women of different body compositions, ranging from underweight to obese (27). The data were extracted from the publication using WebPlotDigitizer (28). We fitted Equation (2) below to these data to estimate blood volume *V_B_* (ml/kg BW) using the patient’s sex, height, and body weight. The fitted *V_B_* (ml/kg BW) curve is plotted against data in **Figure 3** **A** in *Results*. Our equations for *V_P_* and *V_B_*, converted to liters (L), are:


(1) $$V_{P}=V_{B}\left(1-HEM\right)$$

(2) $$V_{B}=a{\left(100+\;{\text{Δ}}_{iBW}\right)}^{n-1}BW$$

where *a* = 1.27, *n* = 0.373 are the fitted constants for *V_B_* (in liters), *HEM* denotes hematocrit—approximated as 0.4 for female patients and 0.45 for male patients, and Δ*_iBW_* is the % deviation from ideal BW (27), a term used for historical rather than normative reasons. Since obese patients have less *V_B_* per kg compared to thinner patients, total *V_B_* per kg increases inversely with BW. Let $\tilde{iBW}\left(H,sex\right)$ be the ideal weight of a patient, with *H* being an individual’s height (meters). Then, the % deviation from ideal weight relative to ideal weight (27) Δ*_iBW_* is:


(3) $$\Delta_{iBW}=100\;\frac{BW-\;\tilde{iBW}\left(H,sex\right)}{\tilde{iBW}\left(H,sex\right)}$$

(4) $$\tilde{iBW}\left(H,sex\right)=\begin{array}{l} 176.3-220.6H+93.5H^{2}\left(male\right)\\ 145.8-182.7H+79.55H^{2}\;\left(female\right) \end{array}$$

Since THYROSIM was initially calibrated against the dataset in (21), we need to ensure that our *V_P_* adjustments in Equations (1)–(4) remain consistent. Based on these formulas alone, the predicted *V_P_* = 2.7 L is substantially lower than the original THYROSIM *V_P_* = 3.2 L for a normal weight patient. Therefore, we scaled the proposed *V_P_* according to a ratio of 3.2 and a reference plasma volume:


(5) $$V_{Pnew}=\;\frac{3.2V_{P}\left(sex,BW,H\right)}{V_{Pref}}$$

where *V_P_* is calculated using Equation (1). To keep the behavior of the model the same for normal weight patients, we chose the reference volume *V_Pref_* so that *V_Pnew_* ≈ 3.2*L* for patients with similar characteristics to those in (22), which had equal numbers of male and female patients of normal weights. With this in mind, we define the reference volume $\frac{\;V_{P}\left(M,BW_{Mref},H_{Mref}\right)+\;V_{P}\left(F,BW_{Fref},H_{Fref}\right)}{2}$ as the average between the predicted plasma volumes from Equation (1) for a reference normal weight male (M) and female (F) patient. The parameters *H_Mref,_ H_Fref,_ BW_Mref,_, BW_Fref_* are fitted to data.

The TSH distribution volume *V_TSH_* in plasma and tissue spaces was estimated as a constant 5.2*L* in the original THYROSIM. More generally, the TSH distribution volume can be expressed as *V_TSH =_ V_P_* + *V_T_* where *V_T_* is the volume of non-vascular tissues where TSH is distributed. We know that *V_P_* must change according to Equation (5) above. If we assume, as a first approximation, that *V_T_* remains the same, we can write


(6) $$V_{TSHnew}\;=\;5.2+\left(V_{Pnew}-3.2\right)$$

In summary, given an individual’s *BW* (kg), *H* (m), and sex (*M,F*), we compute the adjusted plasma volume *V_Pnew_* and adjusted TSH distribution volume *V_TSHnew_* in p-THYROSIM using Equations (1)–(6) with default HEM values 0.45 for male patients and 0.4 for female patients.

In the original THYROSIM model, irreversible T3 clearance is assumed to occur primarily in rapidly exchanging (FAST) compartments (liver and kidneys), with fractional rate *k*_05_. We incorporate the effects of body composition on thyroid hormone clearance by scaling this parameter according to patient BW. We do this separately for male and female patients using allometric scaling:


(7) $$k_{05,\text{new}}=\;\{\begin{array}{ll} C_{\text{M}}k_{05}{\left(\frac{\text{BW}}{{\text{BW}}_{\text{Mref}}}\right)}^{\frac{3}{4}} & \left(\text{male}\right)\\ k_{05}{\left(\frac{\text{BW}}{{\text{BW}}_{\text{Fref}}}\right)}^{\frac{3}{4}} & \left(\text{female}\right) \end{array}$$

### Adjustments to the TSH Brain-Pituitary Submodel

Building on the original THYROSIM, our submodel for TSH secretion is necessarily a quasi-mechanistic input–output model representation—for lack of sufficient mechanistic data in brain. TSH secretion is driven by TRH and dual suppressor inputs—plasma T3 and T4 concentrations, all represented as a harmonic oscillator damped by T3 signals in pituitary and various brain regions, some converted from T4 in brain [**Figure 3** in ref (20)]. Not all regions and pathways are known for all such T3 signals, so we defined a single, lumped variable “brain T3” representing equivalent T3 in all brain regions that affect TSH secretion, directly and *via* indirect intermediate pathways (anterior pituitary, hypothalamus, etc.) (20). We revisit a few quantitative assumptions about hypothalamic and pituitary dynamics in the original THYROSIM model and modify the submodel here.

**Figure 1:**
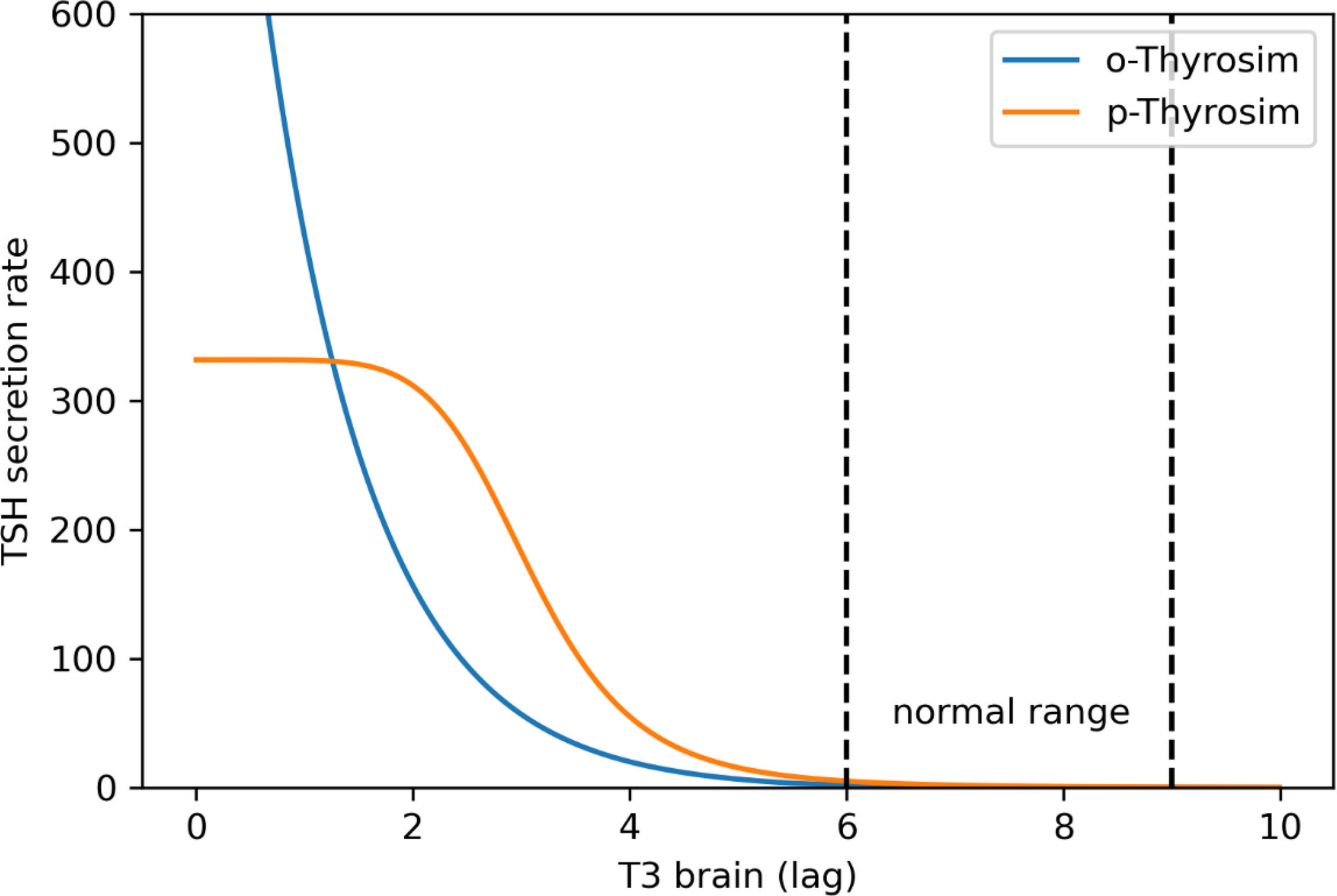
p-THYROSIM (orange) vs. original THYROSIM (blue) TSH secretion rates (μmol/h) versus T3 lag (μmol) in the brain (20). The new TSH secretion function saturates at extremely low T3 values [using Equations (8–10)].

First, in the original model, the TSH secretion function *SR_TSH_* was designed to decrease exponentially as brain T3 increases. This works for normal and mildly hypothyroid patients, but TSH secretion rate in the model becomes unrealistically large when brain T3 falls in this exponential fashion (see **Figure 1**, blue), as we detected in initial attempts to quantify the new model using experimental data based on T3-only treatments (22).

To more accurately reflect TSH secretion as a function of brain T3 levels in completely thyroidectomized patients, we replaced the exponential terms in the original TSH secretion function with Hill functions, such that the new TSH secretion rate matches the old secretion function in the normal range and saturates at extremely low plasma T3, T4, and brain T3 levels. Our updated TSH secretion function is:


(8) $$SR_{TSH}\left(t\right)=\left[B_{0}+\;A_{0}f_{CIRC}\text{sin}\left(\frac{\pi }{12}t-\;\phi \right)\right]\left(\frac{K_{SR_{TSH}}^{m}}{K_{SR_{TSH}}^{m}+\;{\left[T_{3B}^{LAG}\left(t\right)\right]}^{m}\;}\right)$$

(9) $$f_{CIRC}\left(t\right)=\;\frac{{\left[T_{3B}^{LAG}\left(t\right)\right]}^{n}}{{\left[T_{3B}^{LAG}\left(t\right)\right]}^{n}+\;K_{CIRC}^{n}\;}$$

where *A*_0_, *B*_0_ represent the maximum oscillation amplitude and the mean of the TSH secretion rate, respectively, *ϕ* is the phase, *K_CIRC_*, *K_SR_TSH__* are the midpoints for the 2 Hill functions, *m*,*n* are the Hill exponents for the 2 Hill functions, and *f_CIRC_* is a saturating function that modulates the amplitude of the circadian oscillations in TSH secretion according to the patient’s thyroid status. Notably *f_CIRC_ ≈* 0 when T3 in the brain is low, and *f_CIRC_ ≈* 1 when T3 is high. This reflects data that TSH fluctuations cease in extremely hypothyroid patients (29).

Finally, for consistency and better fitting of the model to the data, we also replaced the original equation for *f*_4_ (a nonlinear function that models the transport and conversions of T4 to T3 in brain) with the following Hill function:


(10) $$f_{4}=\;k_{3}\left(1+\;\frac{5K_{f_{4}}^{l}}{K_{f_{4}}^{l}+T_{3B}^{l}}\right)$$

These new Equations (8)–(10) were used in computing the new p-THYROSIM TSH secretion rate curve in **Figure 1**.

### Four Experimental Datasets for Model Quantification and Validation

In addition to the blood volume data from (27), we used 3 published clinical datasets to tune and a fourth to validate our model, as noted earlier. These data contain the T4, T3, and/or TSH responses of male and female patients with various *BW*s and heights (*H*) and varying degrees of thyroid status, subjected to different treatment protocols.

A first dataset, previously used for model fitting to euthyroid patients (19,20), consists of 5-day time course measurements of plasma total T4, T3, and TSH concentrations in 18 healthy men and 18 healthy women volunteers (21). These patients were given 400, 450, and 600 μg of oral T4 doses at hour 24, after a day of fasting. Serial measurements collected over 5 days and averaged at each time point over all male and female subjects together (not individualized) were provided. All patients were reported to have normal BWs and heights. For these simulations, we used *V_P_* = *V_ref_*, an average of a reference male and female patient, as described above, in order to have similar model behavior to the original THYROSIM, fitted using these same data—also as noted above.

A second dataset was used for fitting (tuning) p-THYROSIM parameters to 50 individual hypothyroid patients with no endogenous T4 production (17). Study participants (aged 18 to 65) were from a diverse euthyroid population thyroidectomized for goiter, benign nodular disease, or suspected or known thyroid cancer. All patient data included weight (*BW*) and height (*H*) measurements and gender (*M* or *F*). Following thyroidectomy, patients were prescribed LT4 for replacement therapy. FT4, T3, and TSH measurements were made 1 day before surgery and two post-operative time points (8 and 16 weeks after surgery). For this dataset, we simulated p-THYROSIM for all patient data *individually*, setting the *V_P_* according to the patient’s anthropometric parameters.

A third dataset contained 8-h time course measurements of T4, T3, and TSH individually measured in 18 male and female hypothyroid patients with mixed etiologies, all undergoing LT3 replacement therapy (22). After excluding 3 patients with abnormal TSH behavior, considered to be outliers, we used data from 13 women and 2 men with recorded height, weight, and gender to make the adjustments to the brain–pituitary submodel. These data were used to determine the form of the equations of the brain–pituitary submodel, as described earlier, but was not used in the final parameter fitting.

Finally, a fourth dataset was used for validating the fitted model by comparing p-THYROSIM predicted T4 doses with empirical doses. These data were collected from 554 thyroidectomized patients undergoing LT4 replacement (14) and are featured in other studies (30). Recorded data for individual patients included *BW, H*, sex, starting TSH values, a starting LT4 dose, and an empirical euthyroid dose. The empirical LT4 doses in this dataset were obtained by adjusting the dose until patients achieved a “normal” TSH value between 0.5 and 4.5 mU/L, a typical TSH reference interval. Specific TSH values at the end of the study were not recorded for all patients. We emphasize that this large dataset was not used for fitting the model but instead served as a model validation dataset and, importantly, also used in our dosage prediction experiments.

### Parameter Estimation Criterion

Eleven new model parameters *K_CIRC_*, *K*_*f*_4__ *m*, *n*, *l*, *H_Mref_*, *H_Fref_*, *BMI_Mref_*, *BMI_Fref_*, C_M_ and *K*_*SR*_*TSH*__ in Eqs. (7)– (9) were unknown and optimally estimated from the data. At the same time, we also re-estimated *S*_4_, *k*_05_, *k*_3_, $V_{max}^{\;D1,fast}$ , *A_0_, B_0_=*450 (as detailed in next section) and $V_{max}^{TSH}$ = 0.226 due to a typo in Equation 4 of (31). The first two of these parameters control the intrinsic secretion of T4, the next two control the conversion of T4 to T3 in the brain and slow tissues, while the last two control the mean and amplitude of TSH secretion.

We used a maximum likelihood criterion for parameter estimation. In this methodology, it is assumed that samples from (22,23), and (17) datasets are independent, and further that observed T4/T3/TSH measurements are samples from independent Gaussian distributions with different standard deviations *σ_T_*_4_, *σ_T_*_3_, *σ_TSH_*. If we treat the ordinary differential equation (ODE) solution as the predicted hormone means (µ*_T_*_4_, µ*_T_*_3_, µ*_TSH_*),which are functions of the 17 parameters being optimized, then the total negative loglikelihood for minimization is:


(11) $$LLCostFcn=\sum_{i\;\in \left\{T4,\;T3,\;TSH\right\}}\frac{n\text{log}\left(2\pi \right)}{2}+n\text{log}\left(\sigma_{i}\right)+\sum_{j=1}^{\;n}\frac{{\left(y_{ij}-\;\mu_{ij}\right)}^{2}}{2\sigma_{i}^{2}}$$

where *y_ij_* the *jth* measured value at compartment *i*. Since the variance terms *σ_T_*_4_, *σ_T_*_3_, *σ_TSH_* are unknown, they are also estimated. The parameter values that minimize Equation (11) are the optimum estimates (**Table 1**).

**Table 1:** Comparison of old and new parameter estimates and their %CVs (percent coefficients of variation).

Parameter name	Original THYROSIM estimates	p-THYROSIM estimates	%CV Variability
*S*_4_	0.00174	0.00278	7.90
$V_{\text{max}}^{\text{D}1,fast}$	0.00999	0.0121	1.51
k_05_	0.207	0.185	10.7
*A*_0_	581	220	0.456
*B*_0_	1,166	450	NA
*k*_3_	0.118	0.0589	10.3
K_circ_	NA	3	45.5
*K*_SR_TSH__	NA	3.1	25.6
*n*	NA	5.68	10.1
*m*	NA	6.29	40.3
*K*_*f*_4__	NA	8.5	53.3
*l*	NA	14.4	7.07
*BMI_Mref_*	NA	21.8	4.58
*BMI_Fref_*	NA	23	4.35
*H*_Mref_	NA	1.76	8.42
*H*_Fref_	NA	1.67	4.19
*C*_M_	NA	1.05	3.03

**Figure 2:**
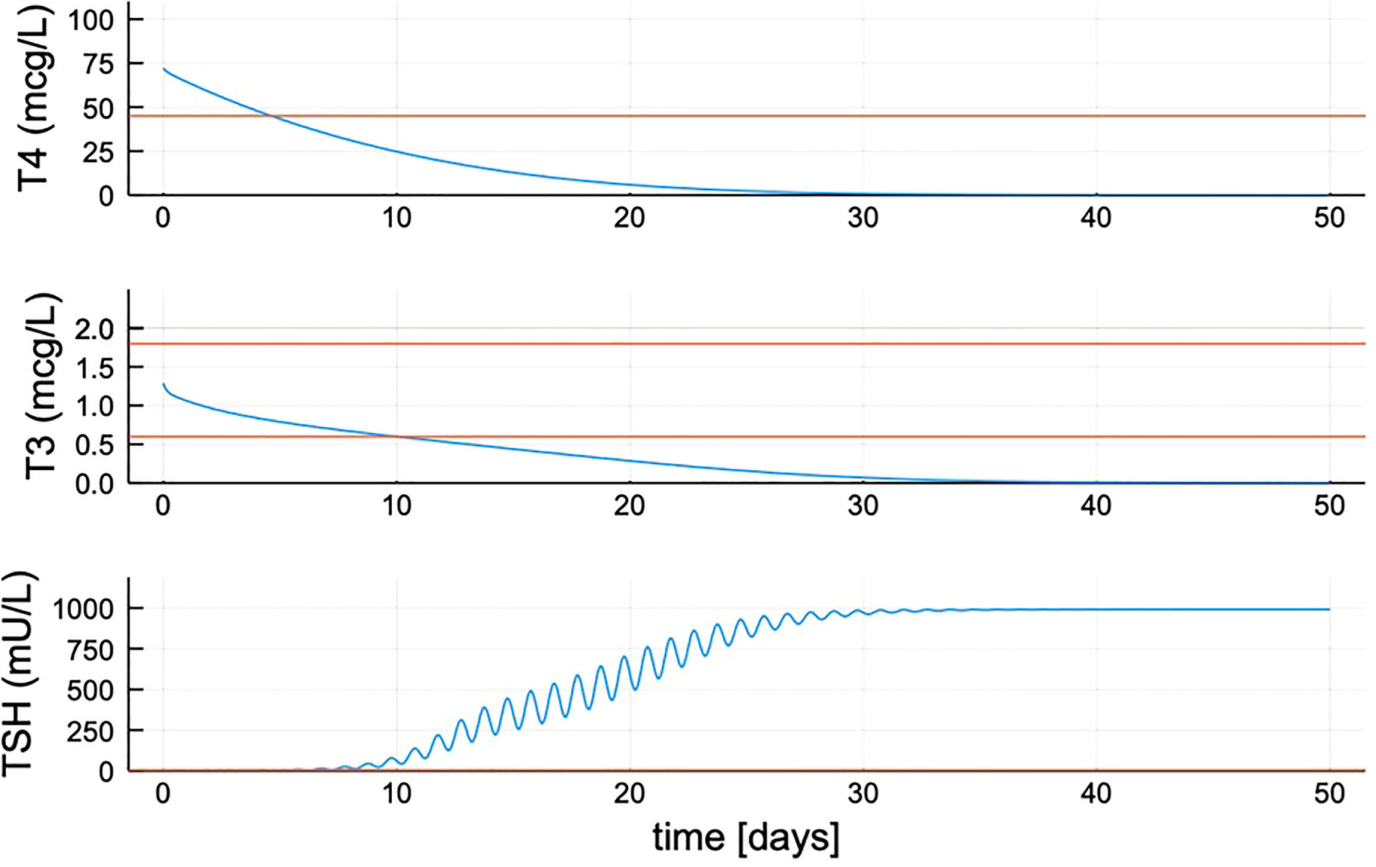
Quantified new model hormone concentration transient responses after simulated thyroidectomy at time zero. After updating the TSH secretion function, TSH peaks approximately 1,000 mU/L. Red lines shown are the minimum for T4 (max ≈ 105) and max and min range for T3 and TSH.

Parameter names are defined in the Methods section and Refs 17–19. The first six parameters are original THYROSIM parameters re-estimated with the updated model equations. The remaining parameters are introduced in p-THYROSIM and are featured in equations (6)– (10) in the text. Here, B_0_ is fixed at 450, resulting in a maximum TSH value of approximately 1,000 mU/L (**Figure 2**). Note that the male and female reference body weights can be obtained from these parameters through the formula ${\text{BW}}_{\text{sex ref}}={\text{BMI}}_{\text{sex ref}}\times H_{\text{sex ref}}^{2}$ . *NA, Not Applicable*.

Finally, to allow the algorithmic search for optimum parameters to capture the TSH fluctuations in the data, we needed to scale some of the data. To accomplish this, we scaled the 4 points that correspond to the two highest and two lowest in the initial TSH trajectory, i.e., the 9th, 13th, 24th, and 28th TSH observations from the euthyroid dataset (21) by a factor of 100.

### Parameter Estimation Search, Best Estimates, and Estimate Variabilities

We searched and found optimum parameter estimates using the gradient-free Nelder-Mead search algorithm implemented in the Julia package Optim.jl (32). Results are shown in **Table 1**. To obtain statistical variabilities for the parameter estimates, we ran a separate fitting routine—Newton’s method (NM), starting at the optimum parameter estimates found with the Nelder-Mead search. NM provides inverse Hessian estimates of the covariance matrix for the estimated parameters, from which primary statistics for parameter optimization were obtained. We report these in **Table 1** as % coefficients of variation (%CVs) for the parameters.

One function in particular was difficult to estimate precisely, the maximum TSH value attainable in untreated hypothyroid patients, which is determined by parameter *B*_0_ in Equation (7) for the TSH secretion rate. We did not have individual patient data for TSH > ~300, and could not find any >500 (the maximum dilution value in many TSH assays). Thus, we fixed the maximum TSH value to a number of plausible values {300, 500, 750, 1000} mU/L (33) by sequentially adjusting the value of *B*_0_ in Equation 7 to accommodate these max TSHs. The result for max TSH = 1000 is *B*_0_ = 450 given in **Table 1**. For the other max TSH values, parameter %CVs agreed with this result to 2 decimal places, so we made maxTSH 1000 in p-THYROSIM (also see **Figures 1**, **2**).

### Dose Prediction

To predict dosing given a patient’s height, weight, and sex, simulations with the individualized parameters for each patient were run using p-THYROSIM. The individual patient’s T4, T3, and TSH trajectories are simulated for 42 days [to mimic the way the data were measured in our large validation dataset (14)]. Patients are assumed to be completely thyroidectomized and model thyroidal secretion rates were thus set to zero. A separate simulation is run for each treatment regime and dose. Daily oral LT4 doses are simulated in 12.5-μg intervals, ranging from 62.5 up to 325 μg. When a single TSH value is used as a target, the predicted optimum dose is the dose that yields a final TSH value closest to 1.8 mU/ml. When a combination of T4 and TSH is used as target, we obtain the optimal LT4 dose as the minimum to the equation $\frac{\left|T4-a\right|}{60}\;+\;\frac{\left|TSH-b\right|}{4}$ , T4 and TSH are the final predicted values for T4 and TSH variables in the model, and *a* and *b* are their respective target values. Dividing the predicted difference by 60 and 4 normalizes the difference in scale between the two model variables.

## Results

### Modeling Patient-Specific Changes in Plasma Volume and T3 Clearance

We incorporated patient *BW, H*, and gender (male *M*, female *F*) into p-THYROSIM by modeling their effects on two aspects of the patient’s physiology important for thyroid hormone metabolism: the patient’s plasma volume *V_P_* and T3 clearance rate. First, we considered how blood volume *V_B_* and plasma volume *V_P_* depend on these patient characteristics.

**Figure 3:**
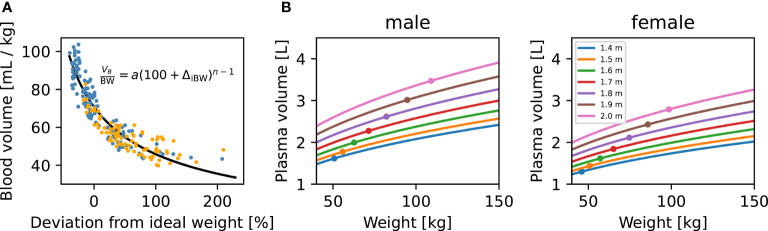
Blood volume (*V_B_*) dependence on body weight (*BW*), height (*H*), and sex. **(A)** Blood volume submodel. Data relating blood volume per mass (27) to the deviation from ideal weight, fitted by least-squares (black curve) with our two-parameter *V_B_* submodel. Individual patients are shown as colored dots (blue: M, orange: F). Our *V_B_* submodel equation is shown inside the panel. **(B)** Model predictions for plasma volume (*V_P_*) of M and F patients. The predicted *V_P_* for male (left panel) and female (right panel) patients are plotted against patient *BW*. Predictions for patients of different heights, ranging from 1.4 to 2 m, are shown as separate curves. Ideal patient weights for the corresponding sex and *H* are shown as dots. For converting *V_B_* to *V_P_*, we assume a hematocrit of 0.45 for male and 0.4 for female patients.

Our blood volume submodel is based on a simple two-parameter equation (**Figure 3A**). Despite its simplicity, this submodel provides a good fit to an experimental dataset consisting of blood volume measurements from 50 male and 50 female patients, as described in (27). This equation was fit to the data in **Figure 3A** separately from the rest of the model in an initial stage. To incorporate our blood volume submodel into the new p-THYROSIM model, we considered the two characteristics height and BMI of hypothetical “reference male” and “reference female” patients whose thyroid hormone dynamics correspond to those predicted by the original THYROSIM model [Equations (3)– (5)]. The height and BMI of these reference patients were considered additional parameters, fit at the same time as the rest of the p-THYROSIM model to the two additional datasets involving time course measurements of thyroid hormones, as described above in *Methods and Data*.

**Figure 3B** illustrates how *V_P_* (in liters) is predicted to change with increasing weight (*BW*) for several example male and female patients of different heights (*H*), according to our blood volume submodel [Equations (1)– (5) in *Methods and Data*]. We note that *V_P_* increases nonlinearly with *BW* and *H* for both genders, showing, for example, why the 1.6 μg/kg dosing formula typically overdoses overweight patients: *V_P_* increases nonlinearly with increasing *BW*, as shown in **Figure 3B**.

Besides changes in *V_B_*, we also expressed T3 fractional clearance rate *k_05_* as increasing allometrically with patient body weight in p-THYROSIM (estimated for females in **Table 1**), with a different scaling for male and female patients, as in Equation 7 in *Methods and Data*. We also updated the brain submodel, so that the TSH secretion rate saturates at a more realistic value in extremely hypothyroid patients, as described in *Methods and Data* and **Figures 1**, **2**.

### New Model Fitted to Data

**Figure 4:**
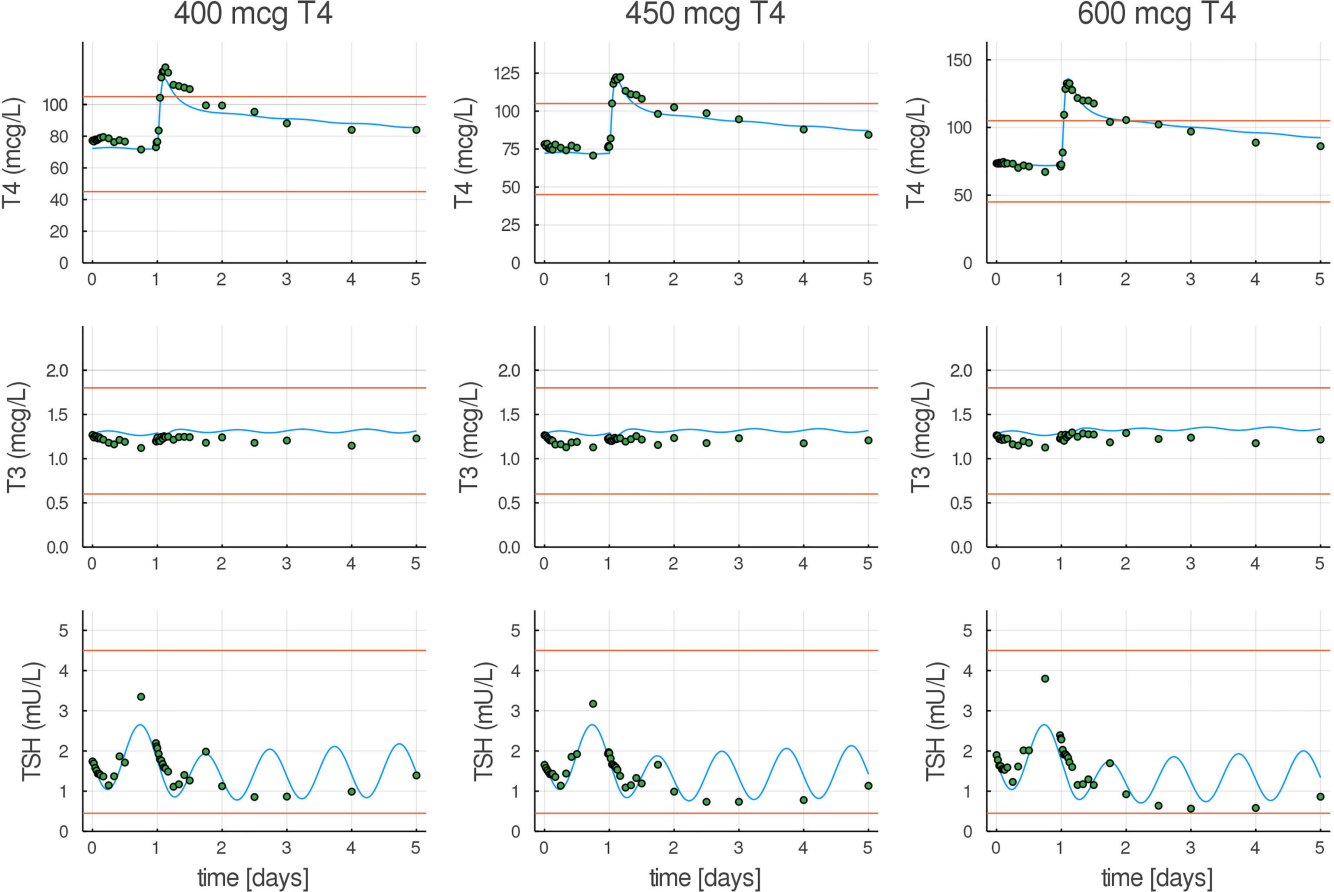
Model predictions compared to T4, T3, and TSH experimental euthyroid data. T4, T3, and TSH responses in healthy volunteers to three different oral doses (400, 450, and 600 µg) of LT4 (21), shown as dots, compared with our fitted model (blue curves). Normal ranges are shown as red lines.

Next, we show that structural changes introduced in p-THYROSIM still give a good fit to euthyroid data previously used to calibrate the original THYROSIM model, albeit with changes in some parameter values. **Figure 4** compares p-THYROSIM model outputs to T4, T3, and TSH time series data, given various T4 oral doses on day 1 (19,21). All model output predictions match the data well. The patient data simulated here are a female of *H* = 1.67 m and *BW* = 63 kg, translating to a BMI of 23, our reference euthyroid normal-weight female patient for p-THYROSIM.

### Model Predictions From Validation Data

Next, we show that p-THYROSIM produces clinically relevant LT4 dosage predictions for thyroidectomized patients from data not used to train it (14), by estimating daily LT4 doses needed to restore normal TSH values (0.5–4.5 mIU/ml). Results are shown in **Table 2**, which also compares the p-THYROSIM model predictions with those determined by the Poisson regression formula in (14), the BMI-based formula in (34), and the standard 1.6 μg/kg BW dosing formula. Notably, p-THYROSIM achieves the highest accuracy in every BMI category.

**Table 2:** LT4 dose predictions classified into BMI categories for p-THYROSIM and 3 other LT4 dosing schemes.

Model	BMI ≤ 26	26 < BMI < 32	BMI ≥ 32	Overall accuracy	Notes
p-THYROSIM	0.63	0.45	0.48	0.53	Dose calculated based on *BW*, *H*, and gender*.
Zaborek et al. (14)	0.50	0.44	0.37	0.44	Dose calculated from a Poisson formula based on *BW*, age, sex, BMI, preoperative TSH, iron, and multivitamin supplements
Papoian et al. (33)	0.52	0.41	0.36	0.43	Dose calculated based on BMI (= *H^2^/BW*)
1.6 mcg/kg	0.52	0.38	0.22	0.38	Dose calculated based on *BW* only

*Dose predictions using p-THYROSIM (run dynamically, over 42 simulation days) correspond to a simulated LT4 dose achieving a steady-state TSH value of approximately 1.8 IU/L (see text for explanation of the computations).

Compared with the 3 other dosing methods, the accuracy of p-THYROSIM optimized dosages for LT4 monotherapy was better overall (53% vs. 44%, 43% and 38%) and for extreme BMI patients (63% vs. ~51% low BMI, 48% vs. ~36% and 22% for high BMI).

To evaluate the performance of competing models, we applied each dosing scheme to individual patient data from Zaborek et al. (14). We then compared the predicted dose to the patient’s euthyroid dose and report the proportion of correctly predicted doses by BMI categories. Finally, we report the proportion of correctly predicted doses for each BMI category across all methods.

### Validating and Predicting LT4 Dosing

**Figure 5:**
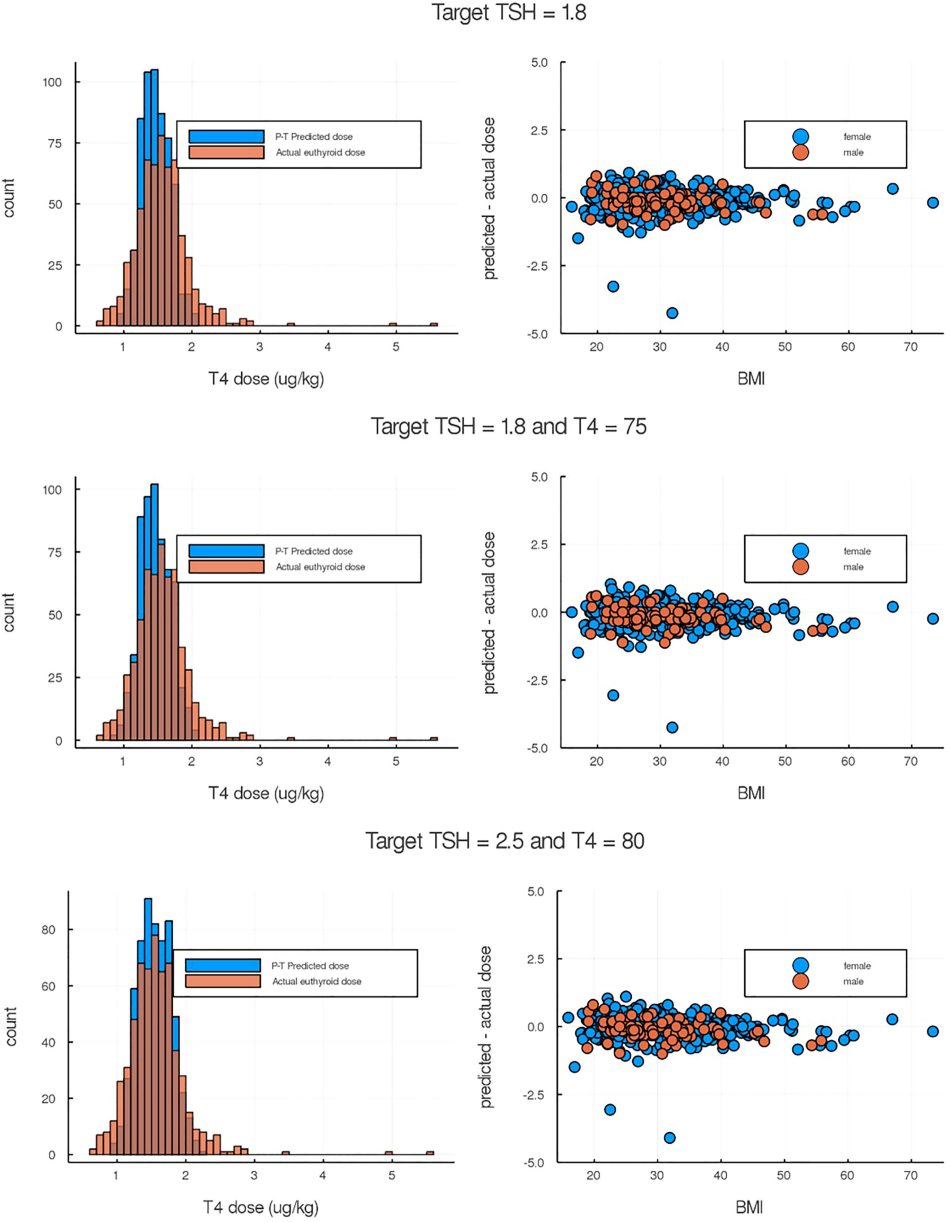
Comparison of p-THYROSIM simulated results against data not used in model fitting. Left-side plots are the histogram distributions of predicted LT4 dose compared to the true euthyroid dose from individual patient data (14). Right-side plots are the differences between predicted and euthyroid doses as a function of BMIs. (Top) p-THYROSIM prediction based purely on TSH criteria (1.8 mIU/ml as target). (Middle and Bottom) p-THYROSIM predictions based on both TSH and T4 measurements.

We explored p-THYROSIM simulated results for more than 500 thyroidectomized patients, undergoing three types of simulated LT4 monotherapy treatment, using anthropometric and dose data from (14). We first considered using only TSH for dosing prediction, as in the data. Then, we numerically explored the simultaneous use of both T4 and TSH measurements for dosing, by simulation experiments, to see if the additional T4 measurements help. Three simulated treatment regimens, corresponding to a TSH measurement-only criterion and two different TSH+T4 regimens, are plotted against validation data from (14) in **Figure 5**.

### Predicting T3+T4 Combination Therapies

**Figure 6:**
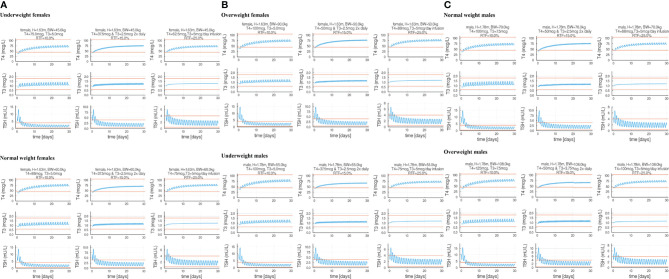
Eighteen combination therapy p-THYROSIM simulations for different residual thyroid function (RTF) values, representing averages of the RTF range computations in reference (26). Each row features a male or a female patient receiving a single LT4 + LT3 combination dose per day (left), two separate LT4 + LT3 combination doses 2× per day (center) and 1× dose of T4 per day combined with constant (ideal therapy) T3 infusion (right). Panel **(A)** includes underweight and normal weight female patients. Panel **(B)** includes overweight female patients and normal weight male patients. Panel **(C)** includes normal and overweight male patients.

Combination oral T3 and T4 therapy dosing can be of interest to clinicians treating patients with unresolved symptoms while taking LT4. We used p-THYROSIM to simulate combination therapies for patients with different anthropometric parameters, levels of RTF (26) and T4+T3 doses. Results of 18 different simulated experiments, including patients of both sexes with various BMI ranges, are plotted in **Figure 6**.

T4 and T3 doses were adjusted incrementally until predicted T4/T3/TSH values are near the midpoints of the normal ranges. These results expand on our previous combined dose predictions using the original THYROSIM, for mixed male–female 70-kg simulated patients classified into three different RTF ranges: RTF <10%, 10%–20%, and >20% (26). Computations for the same ranges were done here with p-THYROSIM, shown as averaged results for the following: combined once a day T4+T3 dosing using RTF = 10% (left-hand sided plots), twice a day T4+T3 dosing using RTF = 15% (MIDDLE plots), and, in the right-hand sided plots, using RTF 25% and once a day T4 dosing + constant infusion T3 dosing—simulating slow-release tablets.

### Residual Thyroid Function in Untreated Male vs. Female Patients for 3 BMI Ranges Predicted From T4 or T3 or TSH Measurements

**Figure 7:**
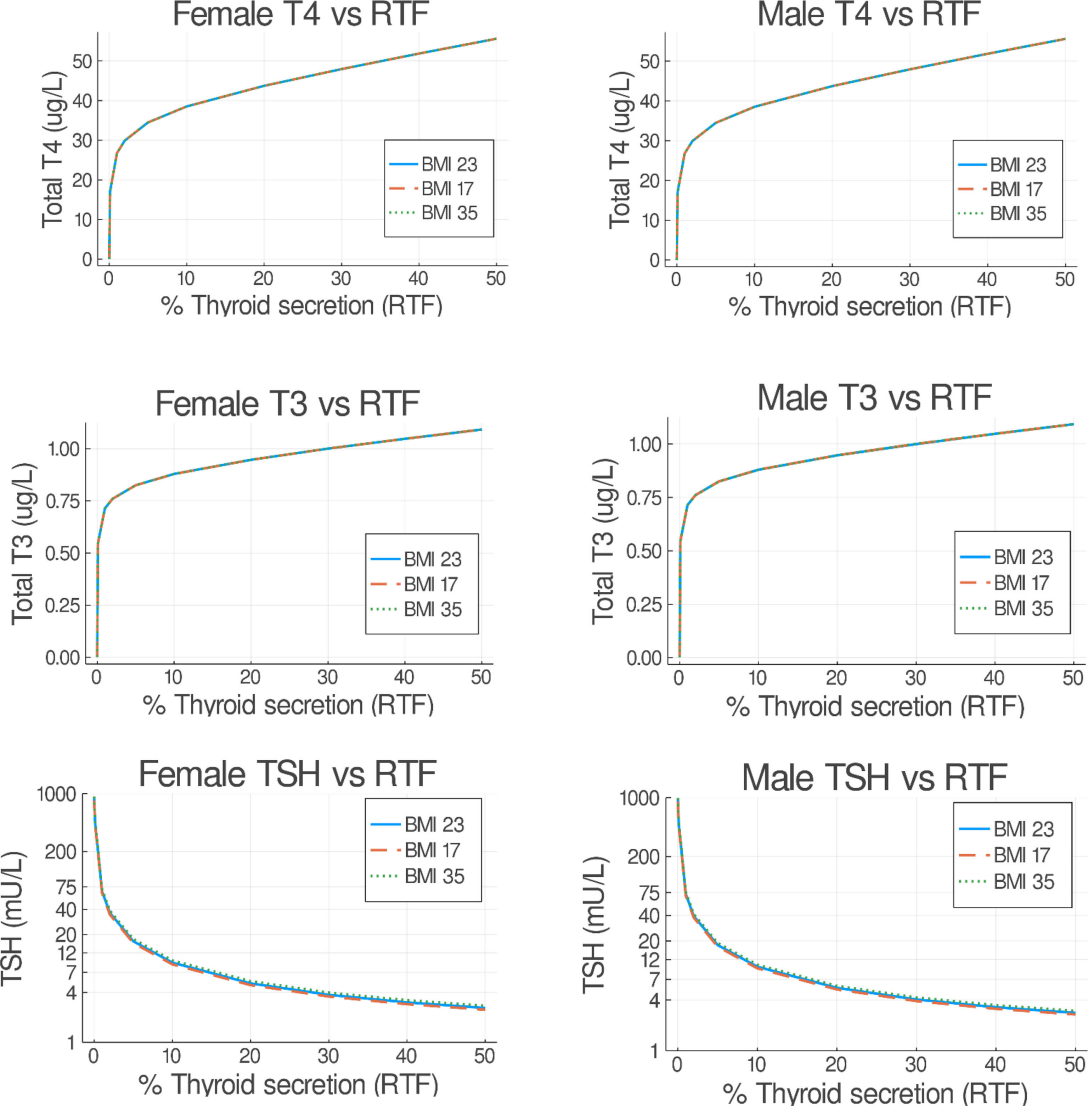
Predicted steady-state serum T3/FT4/TSH concentrations versus residual thyroid function (RTF) values. Untreated underweight, normal, and obese patients were simulated for 50 days. The final steady-state T4/T3/TSH simulation values (as in **Figure 3**) are shown on the abscissa for each RTF value on the ordinate axis, separated into female (left) and male (right) plots. Notably, all resulting BMI plots overlap.

Following the approach in (26), we evaluated RTF values in our patient population, to provide a better basis for replacement therapy. The optimal LT4 dose is influenced by the severity of hypothyroidism, which is therefore dependent on the degree of RTF. Lower values of RTF are typically accompanied by higher TSH and lower thyroid hormone levels, requiring higher doses of LT4. The goal of replacement therapy in (26) was to achieve normal mean steady-state hormone levels for normal weight patients. In this update, we ran p-THYROSIM for 50 simulation days, more than enough time to achieve steady state (see **Figure 2**), reducing thyroidal secretion rates to achieve the different RTFs. We did this for untreated underweight, normal, and obese patient data, separated by gender. Simulated male and female patients had fixed heights of 1.78 m and 1.63 m, respectively. Our results, shown superimposed in **Figure 7**, predict steady-state plasma T4/T3/TSH concentrations for RTF values up to 50%. It is notable that there are no differences in predicted RTFs for the different patient BMI groups, and only TSH vs. RTF results differ very slightly for male patients vs. female patient data; i.e., all have the same simulated steady-state serum concentrations of T3, T4, and TSH for any RTF.

## Discussion

Several decades ago, we reported on using an optimal control theory approach to T4 mono- and T4+T3 combination therapies in typical hypothyroid patients, using two different optimality criteria—restoration of normal hormone levels (i) as quickly as possible and (ii) sigmoidally, to reduce rapid transients (35). The basic model in that work evolved into THYROSIM, our physiologically based and mechanistic simulation model of average human thyroid hormone (TH) regulation (18), both without accounting for sex or anthropometric differences. In this work, we extend the accuracy and applicability of THYROSIM, developing it into p-THYROSIM, for representing TH dynamics in individual patients and more accurately predicting outcomes of clinical interventions. We achieved this by refining mechanistic aspects of TSH regulation and T3 metabolism in the model, by rendering it more consistent with published data, and incorporating commonly measured anthropometric characteristics of individual hypothyroid patients. The primary clinical goal in this regard was to adjust and augment the model so that it can more accurately predict TH dosing requirements in individual patients. [p-THYROSIM is implemented as an efficient open source software package, Thyrosim.jl, in the *Julia* programming language (36) (https://github.com/biona001/Thyrosim.jl). Installation instructions are included with the software package].

In hypothyroid patients, thyroid hormone regulation, absorption, and metabolism can be affected by individual patient factors that can influence individual dosing requirements for achieving euthyroidism. In addition to gender and BMI, age, comorbidities, drug, and supplement interferences, for example, can complicate the problem of optimal dosing. We addressed the two generally considered major factors in our studies, gender and BMI. Importantly, the datasets we used for model quantification and validation (14, 17, 20–22, 27, 30) came from patient populations carefully controlled for many of the other factors.

p-THYROSIM explicitly incorporates patient weight (*BW*), height (*H*), and gender (*M/F*) as model parameters. By incorporating documented effects of these patient characteristics on blood volume and T3 clearance rate, p-THYROSIM provides a means to simulate T4/T3/TSH dynamics in male or female patients with different BMIs. We estimated model parameters using three distinct datasets, and validated model performance and predictive ability using a fourth independent dataset.

In **Table 2**, we report that the finely tuned p-THYROSIM achieved much better dose prediction accuracy than the conventional basis for LT4 dose determination, and better than two other reported computational algorithms (14, 33), especially for patients who have low or high BMI. Overall, dosage predictions using p-THYROSIM appear to be reasonably well-calibrated for male and female patients with varying BMIs, and somewhat more accurate for female than for male patients, outperforming all three alternatives we considered in our comparison. However, this may be due to the higher proportion of female patients present in the datasets we used for model quantification.

In computing the accuracy values in **Table 2**, we realized that accuracy criteria did not align in the referenced studies (18, 33) and are thus not directly comparable with each other. To render them comparable, we established a dose prediction as accurate if it was within 12.5 μg of the clinically determined dose for a patient, and we compared the performance of p-THYROSIM with the other methods using this same criterion. For this reason, the accuracies we report in **Table 2** differ from those originally published, in part because they used different criteria, and possibly also because of the different patient populations studied.

To demonstrate the efficacy and clinical utility of the p-THYROSIM model, fully quantified from several diverse data bases as described in *Methods and Data* and *Results*, we applied it to optimal dosing of both T4-only and combination T4+T3 therapies. A major advantage of mechanistic modeling is its ability to represent and simulate such hypothetical scenarios. p-THYROSIM simulations readily provide the trajectories of plasma T4, T3, and TSH concentrations over time in male or female patients of different height, weight, and degrees of RTF undergoing replacement therapy. We exercised p-THYROSIM (1) to optimize T4-only dosing of hypothyroid patients, using only TSH measurements (2); to investigate whether monitoring T4 as well as TSH may better predict T4 dosing to normalize thyroid function in thyroidectomized patients (3); to evaluate the efficacy of T4+T3 combination therapies compared to traditional T4-only replacement therapies; and (4) to predict how much RTF remains in untreated hypothyroid patients from T3 or T4 or TSH measurements in these patients before they begin treatment.

To address goals (1) and (2) above, we assumed that normal thyroid physiology is based largely on maintenance of normal TH as well as TSH levels in blood, and we tested whether using normal T4 and TSH together as a marker for euthyroidism might have advantage over using TSH alone. Our results in **Figure 5** (top row vs. middle and bottom rows)—comparing dosing based on measurement of TSH-only vs. TSH+T4, are inconclusive. The dataset we used for comparison of these predictions consisted of thyroidectomized patients for which the “correct” L-T4 dose was clinically determined by adjusting the dose until a TSH-only criterion was satisfied, and this may have biased the results.

Our combination therapy study simulations, depicted first in **Figure 6**, predictively illustrate how particular T4/T3 combination therapies might effectively restore normal thyroid function in hypothyroid patients in men and women with different BMIs and different RTFs. RTF is a numerical measure of reduced thyroidal secretion, also computed from the finalized simulated p-THYROSIM model, based on model predictions of serum T3, T4, and TSH levels in patients who have not yet had any replacement therapy (26). We note that the hormone vs. RTF results shown in **Figure 7** are somewhat different than those reported for mixed male–female 70-kg humans using the original THYROSIM (26), not surprising given that the model has been updated in the several ways described in this work.

The graphs in **Figure 6** also confirm quantitatively what is anticipated, that twice daily dosing, or slow-release preparations (which we simulate as constant infusions) result in reduced T3 fluctuations as compared to daily dosing. Our results predict daily optimal T3 doses in combination therapy of 5 to 7.5 μg of T3 combined with 62.5–100 μg of LT4 for women or 75–125 μg of LT4 for men, supporting the concept that not much LT3 is needed in addition to LT4 to restore euthyroid levels. Notably, these amounts are very similar to amounts recommended in guidelines, such as 87.5 μg of LT4 and 6.25 μg of LT3 (35), but may be lower than LT3 doses used by some physicians in clinical practice. That some of these simulation results may deviate from clinical practice suggest opportunities for further research exploring combination therapy with lower T3 doses and slow-releasing T3 formulations. All doses simulated and shown can be achieved using commercially available LT4 and LT3 tablets.

Surprisingly, we note that, in **Figure 7**, neither BMI nor gender has any effect on RTF predictions. Perhaps, this is why these dosage values are not very different from those predictively reported for the 70-kg person in (26). One might hypothesize that, as the thyroid gland fails, this is consistent with the concept that maintenance of thyroid hormone levels by TSH stimulation and TH axis feedback is so heavily ingrained (and dynamically regulated) in normal physiology that these defenses outweigh differences based on gender and BMI.

To conclude, we believe that predictive simulation models like p-THYROSIM and other predictive modeling tools (37) can provide results that improve the efficacy of and shorten if not eliminate the delay in achieving euthyroidism seen with some empirical approaches used in clinical practice (38). For example, they can reduce if not eliminate trial-and-error weight-based practices by clinicians in prescribing T4 or T4+T3 dosing, which likely prolong the time to reach normal steady-state serum hormone levels in patients. The relatively low-dose T3 amounts demonstrated in the **Figure 6** combination therapy predictions can help reduce overdosing with T3 in combination therapy by providing evidence for dosing that can be supported based on normal physiology. We believe that combination therapy should be reserved for patients who do not respond to LT4 with amelioration of their symptoms. Each patient should be treated on an individual basis and the benefits versus risk of such therapy be discussed on an individual basis.

## Software Availability

The p-Thyrosim software is available at https://github.com/biona001/Thyrosim.jl, and the code for parameter estimation is available at https://github.com/biona001/Thyrosim.jl/blob/master/notebooks/fit_all.ipynb.

## Data Availability Statement

The original contributions presented in the study are included in the article; further inquiries can be directed to the corresponding author. Data for parameter estimation is available upon request from the second author, BC.

## Author Contributions

MC-L provided substantial contributions to the modeling, including updates of the TSH secretion submodel and allometric scaling of some parameters, and a first draft of the manuscript. BC provided substantial contributions to the modeling and major computational and programming efforts for this research. Both JJ and DS provided patient data, substantial intellectual contributions, references, and manuscript editing for this research work. Senior author JD formulated the problem, first as a graduate course term project, and guided the intellectual content of the systematic mechanistic modeling and clinical applications through completion of the research. He also did much of the writing of the final manuscript. All authors contributed to the article and approved the submitted version.

## Conflict of Interest

The authors declare that the research was conducted in the absence of any commercial or financial relationships that could be construed as a potential conflict of interest.

## Publisher’s Note

All claims expressed in this article are solely those of the authors and do not necessarily represent those of their affiliated organizations, or those of the publisher, the editors and the reviewers. Any product that may be evaluated in this article, or claim that may be made by its manufacturer, is not guaranteed or endorsed by the publisher.
